# Identification and expression analysis of the glycosyltransferase GT43 family members in bamboo reveal their potential function in xylan biosynthesis during rapid growth

**DOI:** 10.1186/s12864-021-08192-y

**Published:** 2021-12-02

**Authors:** Zhen Li, Xinyue Wang, Kebin Yang, Chenglei Zhu, Tingting Yuan, Jiongliang Wang, Ying Li, Zhimin Gao

**Affiliations:** Key Laboratory of National Forestry and Grassland Administration/Beijing for Bamboo & Rattan Science and Technology, Institute of Gene Science and Industrialization for Bamboo and Rattan Resources, International Center for Bamboo and Rattan, Beijing, 100102 China

**Keywords:** *Phyllostachys edulis*, Glycosyltransferase 43, MYB transcription factors, Yeast one-hybrid

## Abstract

**Background:**

Xylan is one of the most abundant hemicelluloses and can crosslink cellulose and lignin to increase the stability of cell walls. A number of genes encoding glycosyltransferases play vital roles in xylan biosynthesis in plants, such as those of the GT43 family. However, little is known about glycosyltransferases in bamboo, especially woody bamboo which is a good substitute for timber.

**Results:**

A total of 17 GT43 genes (*PeGT43*–*1* ~ *PeGT43*–*17*) were identified in the genome of moso bamboo (*Phyllostachys edulis*), which belong to three subfamilies with specific motifs. The phylogenetic and collinearity analyses showed that *PeGT43*s may have undergone gene duplication, as a result of collinearity found in 12 pairs of *PeGT43*s, and between 17 *PeGT43*s and 10 *OsGT43*s. A set of *cis*-acting elements such as hormones, abiotic stress response and MYB binding elements were found in the promoter of *PeGT43*s. *PeGT43*s were expressed differently in 26 tissues, among which the highest expression level was found in the shoots, especially in the rapid elongation zone and nodes. The genes coexpressed with *PeGT43*s were annotated as associated with polysaccharide metabolism and cell wall biosynthesis. qRT–PCR results showed that the coexpressed genes had similar expression patterns with a significant increase in 4.0 m shoots and a peak in 6.0 m shoots during fast growth. In addition, the xylan content and structural polysaccharide staining intensity in bamboo shoots showed a strong positive correlation with the expression of *PeGT43*s. Yeast one-hybrid assays demonstrated that PeMYB35 could recognize the 5′ UTR/promoter of *PeGT43–5* by binding to the SMRE *cis*-elements.

**Conclusions:**

*PeGT43*s were found to be adapted to the requirement of xylan biosynthesis during rapid cell elongation and cell wall accumulation, as evidenced by the expression profile of *PeGT43*s and the rate of xylan accumulation in bamboo shoots. Yeast one-hybrid analysis suggested that PeMYB35 might be involved in xylan biosynthesis by regulating the expression of *PeGT43–5* by binding to its 5′ UTR/promoter. Our study provides a comprehensive understanding of *PeGT43*s in moso bamboo and lays a foundation for further functional analysis of *PeGT43*s for xylan biosynthesis during rapid growth.

**Supplementary Information:**

The online version contains supplementary material available at 10.1186/s12864-021-08192-y.

## Background

As an important nontimber forest product, bamboo is usually characterized by rapid growth, high strength and flexible culms, which are attributed to fast accumulation and elaborate proportions of lignin, cellulose and hemicellulose. Cellulose forms the main structural framework of the cell wall. Hemicellulose covers cellulose microfibrils and crosslinks with them to form a stable network by hydrogen bonding. Lignin is a heterogeneous mixture of randomly polymerized phenolic monolignols that is interspersed and crosslinked with hemicellulose to make cell wall stronger. The biosynthesis of these major cell wall components is regulated by a set of transcription factors, some of which can regulate both cellulose and hemicellulose synthesis as well as lignin synthesis, such as MYB26, MYB46/83, SND1 and KNAT7 [1–4]. Genes associated with lignin, cellulose and hemicellulose biosynthesis in moso bamboo have been identified, such as the members of NAC, MYB, CESA, LAC and UGDH gene families [5–8].

It has been shown that the hemicelluloses of moso bamboo are dominated by xylan [9, 10], which is composed of 1,4-β-D-xylose as the backbone, with glucuronic acid, arabinose and acetylation in the side chains [11]. Glycosyltransferases (GTs) play important roles in the biosynthesis of xylans, such as members of the GT8, GT43, GT47, GT61 and GT75 families. A set of IRREGULAR XYLEM (IRX) genes, *IRX7*, *IRX8*, *IRX9*, *IRX9L*, *IRX10*, *IRX10L*, *IRX14* and *IRX14L* encoding GTs in plants, have been found to be for xylan biosynthesis [12]. The *irx7*, *irx8*, *irx9*, and *irx14* mutants in Arabidopsis (*Arabidopsis thaliana*) exhibited a collapsed xylem phenotype and a significant decrease of xylose in the cell wall, whereas *irx10* showed a moderate degree of xylose reduction. MALDI-TOF MS analysis indicated that the number of glucuronoxylans (GX) chains decreased and that the GX chain length increased in *irx7* and *irx8* plants, whereas the number of GX chains increased and the GX chain length decreased in *irx9* and *irx14* plants, suggesting that *IRX7* and *IRX8* may be involved in the synthesis of a xylan primer, whereas *IRX9* and *IRX14* may be required to synthesize the xylan backbone [13, 14]. Coimmunoprecipitation and bimolecular fluorescence complementation analysis provided biochemical evidence for the xylan synthase complex (XSC) formed by AoIRX9, AoIRX10 and AoIRX14. AoIRX9 plays an important role in the structural properties of the complex, as the activity of AoIRX10 and AoIRX14 is significantly reduced in the absence of AoIRX9 [15]. Coexpression of TaGT43–4, TaGT47–13, TaGT75–3, and TaGT75–4 in *Pichia pastoris* confirmed that these proteins form a complex. Among them, TaGT43–4 acts as a scaffold protein that holds the other proteins [16]. The side chain α-D-glucuronic acid (GlcUA) of xylan was reduced in *irx10* plants, suggesting that *IRX10* was involved in side-chain substitution [17, 18]. *PARVUS* is involved in both pectin and xylan synthesis, while the *FRA8* and *F8H* families act to modulate xylan contents and polymerization [14, 19].

The GT43 family has been reported in many model plants, because all members, such as *IRX9*, *IRX9L*, *IRX14* and *IRX14L*, are involved in the biosynthesis of xylan. The biomass of the RNA interference lines showed higher digestibility, decreased xylan, arabinose and ferulic acid, and increased coumaric acid, supporting a causative role of *BdGT43A* in *Brachypodium distachyon* [20]. The *BdGT43B2* knockout lines showed that the immunofluorescence labeling of xylan was greatly reduced, whereas cellulose labeling was unchanged or slightly increased [21]. Overexpression of *OsIRX9*, *OsIRX9L* and *OsIRX14* in Arabidopsis mutants *irx9* and *irx14* resulted in xylosyltransferase (XylT) activity in the stems that was greater thandouble that in wild type plants, and the stem strength increased to 124% above that of wild-type plants, suggesting that *OsIRX9*/*OsIRX9L*, and *OsIRX14*, have similar functions to Arabidopsis *IRX9* and *IRX14*, respectively [22]. In addition, other GT43 members in rice such as *OsGT43A* and *OsGT43E* can partially complement the defects of *irx9* and *irx14* mutants, indicating their similar functions in xylan biosynthesis [23]. GT43s in cotton and poplar also have similar functions as Arabidopsis IRX genes. Overexpression of *GhGT43A1* and *PoGT43B* in *irx9* rescued the defects in plant size and secondary wall thickness, and partially restored the xylose contents. Moreover, *GhGT43C1* and *PoGT43C* were able to rescue the defect of *irx14* [24, 25].

Transcription factors (TFs) affect the biosynthesis of xylan by regulating GT43 genes. KNAT7 and OSH15 changed the transcript levels of *AtIRX9* and *OsIRX9* in Arabidopsis and rice by binding to their promoters respectively. The *knat7* mutants had lower transcript levels of *AtIRX9*, reduced xylan contents, and showed a lack of xylose immunofluorescence labeling [26]. OSH15 regulates xylan biosynthesis via *OsIRX9* and affects branching angle and plant shape [27]. MYB46 was found to activate the expression of *KNAT7* and *IRX14L* using the estrogen-inducible direct activation system, and SND1 and VND7 also altered the expression levels of *IRX14L* and *IRX10*. Moreover, potential MYB- and NAC-binding elements were identified in the promoters of these genes [28]. The *Miscanthus lutarioriparius* GT43 gene family was also shown to be regulated by MYB and NAC, as MlSND1, MlMYB46 and MlVND7 bound to *MlGT43A* and *MlGT43B* upstream sequences and enhanced the fused GUS activity [29].

To investigate xylan accumulation in moso bamboo during rapid growth, the molecular characteristics and expression patterns of 17 GT43 genes were identified and analyzed in moso bamboo. Furthermore, a predicted regulatory network of *PeGT43*s was constructed. The expression correlation of genes in the network and the regulation between PeMYB35 and the hub gene of *PeGT43–5* were verified. This study will serve as a useful reference for further functional analyses of *PeGT43*s involved in bamboo cell wall development in terms of xylan accumulation and for the future molecular mechanism of GT43 genes in related plant species.

## Results

### Identification of GT43 genes in moso bamboo

The genomes of moso bamboo and other four bamboo species (*Bonia amplexicaulis*, *Guadua angustifolia*, *Olyra latifolia*, *Raddia guianensis*) have been sequenced with moso bamboo genome reaching the chromosome level [30, 31], which enabled genome-wide identification of the GT43 gene for different bamboo species. A total of 24 potential GT43 genes were found in the moso bamboo genome. After screening the conserved domain by CD-Search program from the NCBI, only 17 genes encoding complete glycosyltransferase 43 (GT43) were identified and designated *PeGT43*–*1* ~ *PeGT43*–*17*. For comparison with GT43 genes in other bamboos, the genomes of *O. latifolia*, *R. guianensis*, *G. angustifolia* and *B. amplexicaulis* were also used, in which 7, 6, 6 and 12 GT43 genes were identified, respectively. Detailed information regarding these *GT43* genes such as gene number, protein length and molecular weight is listed in Table S1.

Comparative analysis revealed that woody bamboo had more GT43 gene family members than herbaceous bamboo. The most GT43 gene family members were found in moso bamboo, followed by *B. amplexicaulis*, while *G. angustifolia* had only six complete GT43 genes. In addition, there were 10, 10, 14, 4, 9 and 6 GT43 genes in the genomes of Brachypodium, rice, maize, Arabidopsis, cotton and poplar, respectively, in which the gene number of GT43 was greater in the monocots than in the dicots. The number difference of GT43 gene family members in different species may be related to their xylan biosynthesis requirements, considering the abundance of xylan in monocots [9, 10].

### Phylogenetic analysis of GT43s

**Fig. 1:**
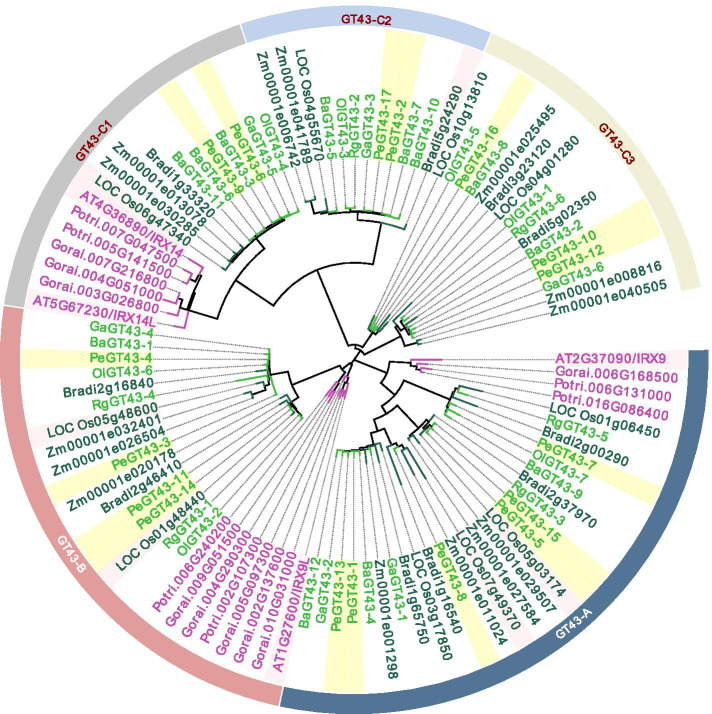
Phylogenetic tree of GT43 family. Phylogenetic tree of GT43s from *Phyllostachys edulis* (Pe), *Olyra latifolia* (Ol), *Raddia guianensis* (Rg), *Guadua angustifolia* (Ga), *Bonia amplexicaulis* (Ba), *Brachypodium distachyon* (Brandi), *Oryza sativa* (Os), *Zea mays* (Zm), *Arabidopsis thaliana* (At), *Gosspium hirsutum* (Gorai) and *Populus trichocarpa* (Potri). Subfamilies of A, B and C are labeled in pink, blue and purple, respectively. PeGT43s and GT43s identified in other species are shaded yellow and pink, respectively; green and red nodes represent monocots and dicots, respectively

To investigate the origin and evolution of GT43s, a maximum likelihood phylogenetic tree was constructed (Fig. 1). All 101 GT43 members from 11 plant species were classified into three subfamilies (A, B, and C), of which the largest one, C, was further divided into three groups (C1, C2 and C3). GT43s from monocots and dicots were clustered into different branches in each subfamily, but the C2 and C3 groups only contained GT43s from monocots. The highest protein homology of GT43s was found between moso bamboo and the other four bamboo species, followed by Brachypodium and rice. There was at least one GT43 homologous protein of moso bamboo compared with those in Arabidopsis and rice. IRX9 (AT2G37090), IRX9L (AT1G27600) and IRX14/IRX14L (AT4G36890/AT5G67230) associated with xylan biosynthesis [22] were clustered in the A, B and C subfamilies, which were close to PeGT43–8, PeGT43–14 and PeGT43–6/PeGT43–9, respectively. PeGT43–15/PeGT43–5 and PeGT43–4 were presumed to be involved in glucuronide xylan synthesis as they were homologous to OsGT43A (LOC Os05g03174) and OsGT43E (LOC Os05g48600), respectively [23]. PeGT43–17/PeGT43–2 were homologs of BdGT43B2 (Bradi5g24290), which is involved in arabinoxylan synthesis [21].

**Fig. 2:**
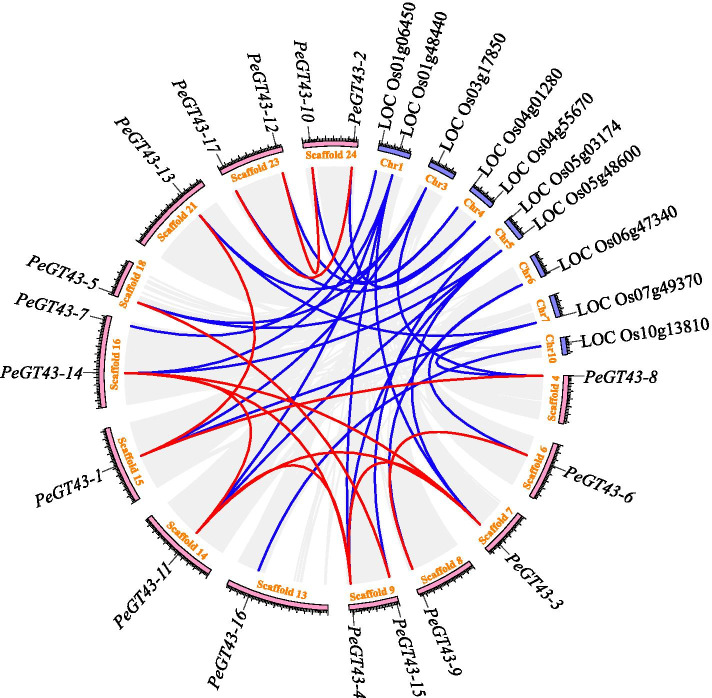
Collinearity of *PeGT43*s and *OsGT43*s. The gray lines indicate duplicated blocks, while the blue and red lines indicate the collinear gene pair of *PeGT43*s and those between *PeGT43*s and *OsGT43*s

To examine the distribution of *PeGT43*s in moso bamboo scaffolds and their evolution, *PeGT43*s and *OsGT43*s were mapped onto moso bamboo and rice genomes for collinearity analysis (Fig. 2). Seventeen *PeGT43*s were located on 13 scaffolds of moso bamboo, of which 15 *PeGT43*s comprised 12 gene pairs. *PeGT43–3*, *PeGT43–4*, *PeGT43–11* and *PeGT43–14* from the B subfamily shared collinearity on different scaffolds, showing evidence of more than one duplication event in moso bamboo. *PeGT43*s were compared to the rice genome with 10 collinear *OsGT43*s. *PeGT43*s (*PeGT43–1*, *PeGT43–3*, *PeGT43–4*, *PeGT43–5*, *PeGT43–6*, *PeGT43–8*, *PeGT43–9*, *PeGT43–11*, *PeGT43–13* and *PeGT43–14*), which were homologs of *OsIRX9*/*OsIRX9L* (LOC Os07g49370/LOC Os01g48440) and *OsIRX14* (LOC Os06g47340) were all found in pairs on different scaffolds and were twice the number of direct homologs in rice. However, *PeGT43–7* and *PeGT43–16* were collinear with LOC_Os01g06450 and LOC_Os10g13810, respectively, suggesting that no expansion occurred in them. Furthermore, the *Ka*/*Ks* ratio of duplication pairs and approximate divergence dates were estimated. The results showed that the duplication pairs underwent purifying selection as the *Ka*/*Ks* values of the duplication pairs were less than 1.0. Duplication events may have taken place from 62.14 million years ago (Mya) to 8.61 Mya (Table S2). The divergence of *PeGT43–4* and *PeGT43–14* in the B subfamily occurred at the earliest time, probably before the divergence of moso bamboo and rice. The majority of gene pairs with collinearity, however, diverged at a late time. According to the potential function of *PeGT43*s, it is believed that the expansion of these genes may be the result of partial gene duplication to adapt to biological function.

### Conserved motifs of PeGT43s

**Fig. 3:**
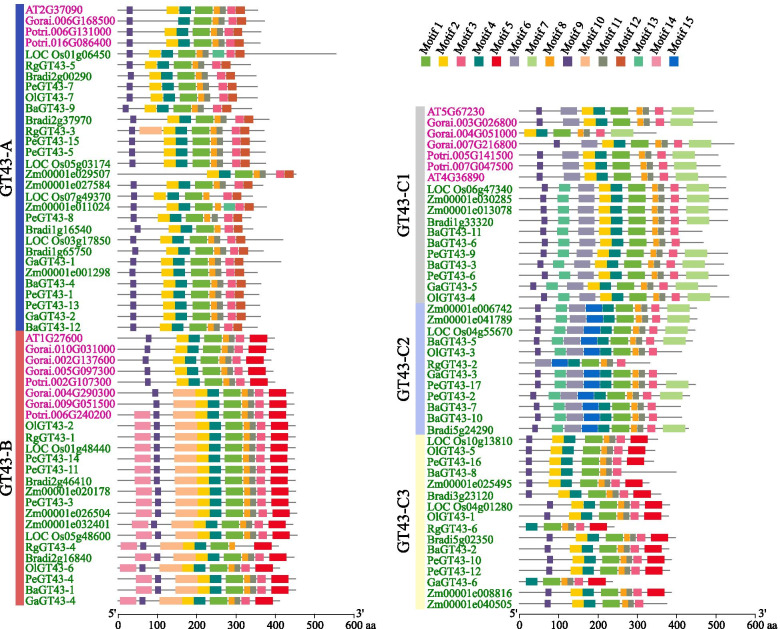
Conserved motifs of GT43s. The length of GT43s is presented as black lines. The boxes in different colors distributed on the black line represent the conserved motifs of GT43s

To further clarify the specific motifs of PeGT43s, the distribution of 15 main conserved motifs was analyzed with the MEME online program (Fig. 3). GT43s in the same cluster possessed similar motifs regarding the number and position, implying that they may be functionally similar. Motif 1, Motif 3, Motif 4, Motif 8, Motif 9 and Motif 11 were shared by all 101 GT43 proteins. Motif 12 was specific to the A subfamily. Motif 5, Motif 10 and Motif 14 were found exclusively in the B subfamily, but those of dicots in this subfamily lacked Motif 10 or Motif 14. Motif 7, Motif 6, Motif 13 and Motif 15 were specific to the C subfamily, of which Motif 15 was found only in the C2 group. In addition, the conserved motifs specific to monocots were Motif 10, Motif 13, Motif 14, and Motif 15.

There were obvious differences in the C-terminus and the N-terminus of the GT43s, which may be related to the recognition of donors and acceptors of GTs. Four of the 15 conserved motifs were reported in crystal structures of GTs. ‘DD[DS]N’ and ‘W[HRNW][LT][RQKH]’ were located within Motif 1 and Motif 14, with amino acid sites ‘DD[DS]N’, ‘[HRNW]’ and ‘[RQKH]’ as donor binding sites. The ‘[EQ][GA]P’ and ‘[ILVM][DEH][MWI][AS][GS]F’ were located within Motif 8 and Motif 3, with amino acid sites ‘[EQ]’ and ‘[DEH]’ as acceptor binding sites [32].

### *Cis*-acting elements in the promoter of *PeGT43*s

**Fig. 4:**
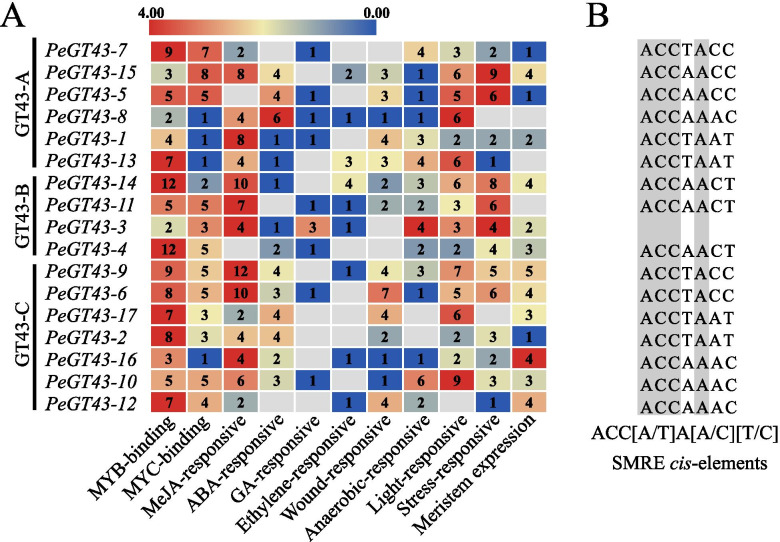
*Cis*-acting elements in the promoter of *PeGT43*s. Upstream sequences (2000 bp) of the *PeGT43*s start codon were selected as the promoters. **A**: Corresponding *cis*-acting elements, the color bar indicates the log_2_-based number of these elements. **B**: SMRE *cis*-elements, the conserved nucleic acid bases are shaded in grey

*Cis*-acting elements distributed in the promoters of target genes affect their expression. Screening for the *cis*-acting elements in upstream sequences of *PeGT43s* revealed a large number of TF binding elements, hormone response *elements and abiotic stress response elements* (Fig. 4 and Table S3). *The most abu*ndant elements were MYB binding elements and MeJA-responsive elements, followed by MYC binding elements, light-responsive elements and stress-responsive elements. In addition, several promoters of *PeGT43*s contained wound-responsive elements, ABA-responsive elements, anaerobic-responsive elements and meristem expression elements. These results suggested that *PeGT43s* may be regulated by many internal factors and respond to environmental changes in moso bamboo.

TFs play an important role in plant growth and development by binding *cis*-acting elements of targeted genes. To investigate the potential transcriptional regulatory mechanisms, the potential regulatory relationships were analyzed by PlantRegMap (Fig. 4 and Table S4). Three hundred and fifteen regulatory relationships between 60 TFs and 17 *PeGT43*s were identified under a cutoff *p value* ≤ 0.05, with MYBs and ERFs being the most enriched TFs. Fourteen *PeGT43*s promoters possessed ERF binding sites and 16 *PeGT43*s promoters possessed SMRE elements binding MYBs. GATA, LBD, C2H2 and NAC binding elements were also found in the promoter sequences of *PeGT43*s.

### Expression profiles of *PeGT43*s in different tissues of moso bamboo

**Fig. 5:**
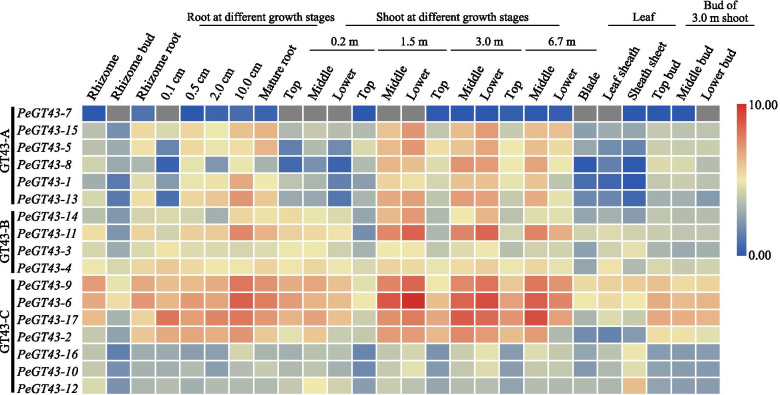
Expression profiles of *PeGT43*s in different tissues of moso bamboo. The color bar indicates log_2_-based fragments per kilobase per million (FPKM). The FPKM value was listed in Table S5

To reveal the potential function of *PeGT43*s, we analyzed the expression profiles of *PeGT43*s in various tissues with RNA-seq data. The results of 17 *PeGT43*s in 26 different tissues showed that most *PeGT43*s were expressed at high levels in roots and shoots, especially in the middle and base portions of 1.5 m to 6.0 m shoots, while *PeGT43–12* was most highly expressed in 0.2 m shoots and sheath sheets (Fig. 5 and Table S5). *PeGT43–7* was nearly absent in all tissues. *PeGT43*s of the A subfamily were expressed mainly in mature roots and shoots but only to a small extentin leaves, while those of the C subfamily showed various levels of expression, not only in the roots and shoots but also in the buds. For example, *PeGT43–5* of the A subfamily exhibited preferential transcriptional abundance in mature roots and the base of 1.5 m and 3.0 m shoots, but *PeGT43–17* of the C subfamily was also exhibited a relatively high abundance in buds.

**Fig. 6:**
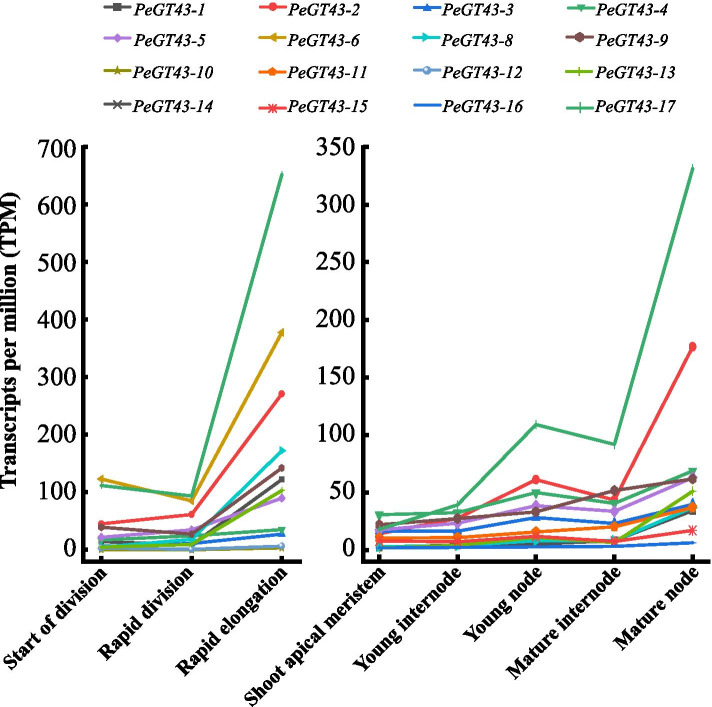
Expression profiles of *PeGT43*s in different portions of moso bamboo shoots. *PeGT43*s with significant transcriptional changes were showed in the folding line chart, using the transcripts per million (TPM) as the value of the vertical coordinate. The TPM value was listed in Table S5

Due to the preferential abundance in shoots, the expression profiles of *PeGT43*s based on RNA-seq data from different growth stages and parts of the shoots were analyzed. The results showed that *PeGT43*s were expressed specifically in shoots of different heights, which was consistent across 26 different tissues (Fig. S1 and Table S5). Furthermore, transcriptional analysis of different parts of shoots at the early stage of rapid growth (3.0 m shoots) was performed (Fig. 6 and Table S5). The expression levels of most *PeGT43*s were significantly different (*p* < 0.05) among the start of division (SD), rapid division (RD) and rapid elongation (RE) sections (Fig. 6 and Table S5). The genes belong to A and B subfamilies were highly expressed in RE section but nearly absent in the SD and RD sections. *PeGT43–2*, *PeGT43–6*, *PeGT43–9* and *PeGT43–17* belonging to the C subfamily were highly expressed in all portions of the 3.0 m shoot, with the highest expression level in the RE section. In addition, the transcripts of *PeGT43–4* and *PeGT43–5* accumulated substantially from the SD to the RE section.

Nodes are considered to be important tissues to provide mechanical support during the rapid growth of moso bamboo. Therefore, the *PeGT43*s expression patterns in the shoot apical meristem (SAM), young internode (YIN), young node (YNO), mature internode (MIN) and mature node (MNO) of shoots were analyzed (Fig. 6 and Table S5). The expression levels of *PeGT43*s were lower in the SAM, and higher in nodes than in internodes, and those in mature tissues were higher than those in young tissues. Twelve *PeGT43*s were differentially expressed at significant levels (*p* < 0.05). *PeGT43–2* and *PeGT43–17* showed much higher expression levels in the nodes than other *PeGT43*s.

### Co-expression network of *PeGT43*s in moso bamboo

**Fig. 7:**
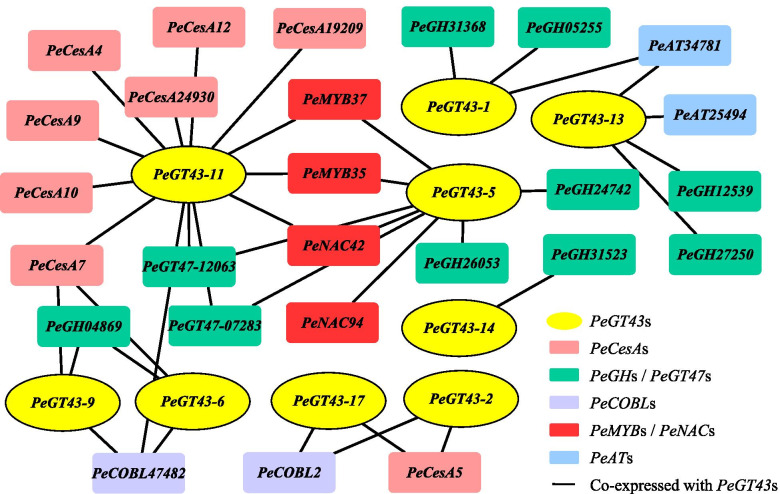
The co-expression network of *PeGT43*s

To determine the genes coexpressed with *PeGT43*s, a coexpression network was constructed using the BambooNET online database. A total of 310 nodes and 1871 connections were found in the network coexpressed with *PeGT43*s. The GO terms showed that the majority of the coexpressed genes were enriched in biological pathways involved in polysaccharide metabolism, such as cellulose biosynthetic process (GO:0030244), cellular polysaccharide biosynthetic process (GO:0033692), beta-glucan biosynthetic process (GO:0051274) and cellular carbohydrate biosynthetic process (GO:0034637), which implied an important function of *PeGT43*s in cell wall reformulation (Fig. S2). The molecular function category showed that the coexpressed genes had UDP-glycosyltransferase activity (GO:0008194) and cellulose synthase activity (GO:0016759). A network containing 35 genes with coexpressed Pearson correlation coefficient (PCC) > 0.8 was created by identifying categories associated with cell wall biosynthesis (Fig. 7 and Table S6). Of 35 genes, four genes were selected as the most significantly coexpressed genes encoding TFs. In addition, multiple genes encoding cellulose synthases (CesAs), glycosyltransferases (GTs), glycosyl hydrolases (GHs), acetyltransferases (ATs) and glycosylphosphatidylinositol-anchored proteins (COBLs) were identified to be significantly coexpressed with *PeGT43*s in the network.

### Expression abundance of *PeGT43*s and co-expressed genes consistent with xylan content

**Fig. 8:**
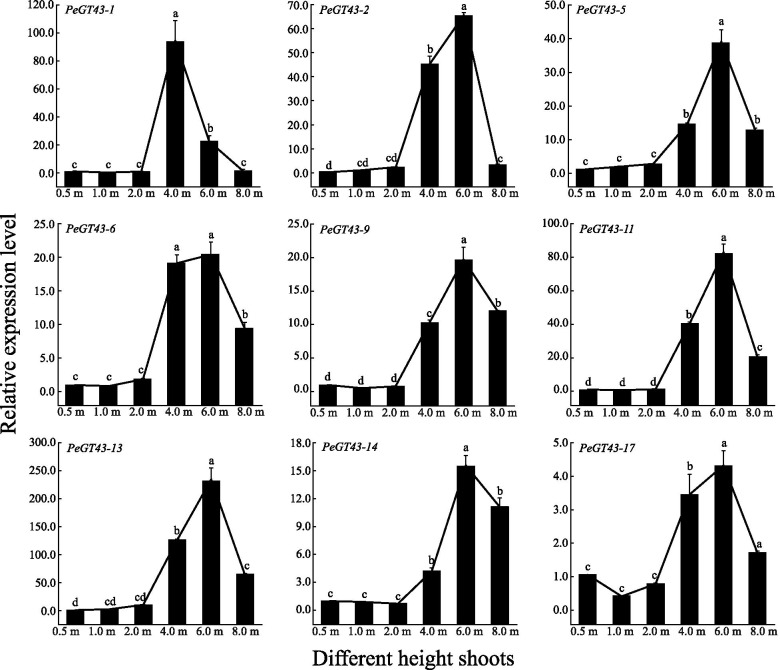
Relative expression level of *PeGT43*s analyzed by qRT-PCR. Lowercase letters a, b, c, d and e indicate significant differences in expression level (*p* < 0.05)

To confirm the correlation between *PeGT43*s and coexpressed genes in shoots of different heights, the expression of 9 representative *PeGT43*s and nine coexpressed genes (PCC > 0.9) within the network was explored in a qRT–PCR assay. The expression level of *PeGT43*s was low at the early growth stages (0.5 m ~ 2.0 m) of the shoots, and then showed a significant increase (*p* < 0.05) when the shoots grew to 4.0 m. The expression of most *PeGT43*s continued to rise significantly (*p* < 0.05) during growth from 4.0 m to 6.0 m and peaked in 6.0 m shoots, while a few *PeGT43*s showed similar expression levels in 4.0 m and 6.0 m shoots. Then, the expression of *PeGT43*s decreased and was maintained at a high level (*p* < 0.05) in 8.0 m shoots (Fig. 8).

**Fig. 9:**
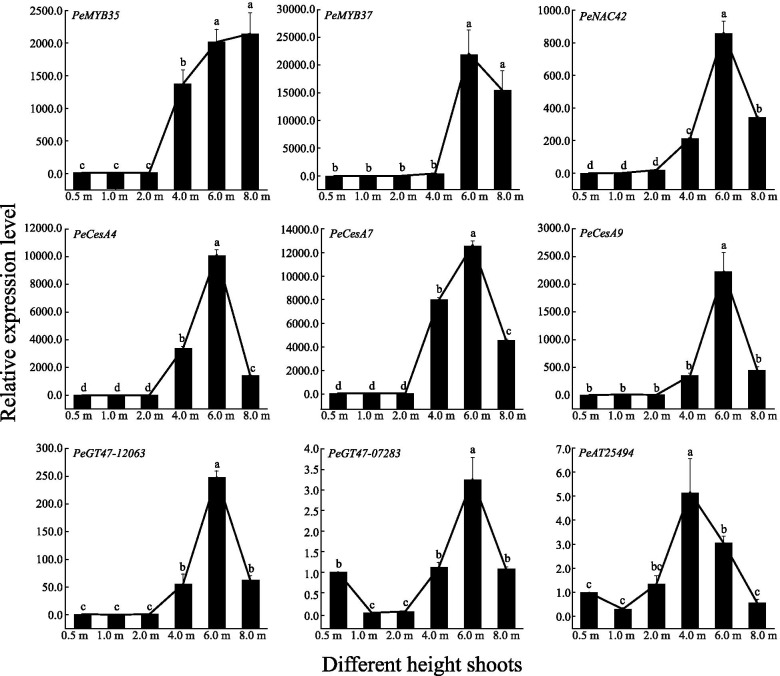
Relative expression level of co-expressed genes analyzed by qRT-PCR. Lowercase letters a, b, c, d and e indicate significant differences in expression level (*p* < 0.05)

The coexpressed genes showed similar expression profiles to those of *PeGT43*s, among which *PeMYB35*, *PeMYB37*, *PeNAC42*, *PeCesA4*, *PeCesA7*, *PeCesA9*, and *PeGT47–12,063* showed great changes in transcription level. Most of them were expressed at low levels at the initial growth stages (0.5 m ~ 2.0 m) but increased significantly (*p* < 0.05) when the shoots grew to 4.0 m or 6.0 m, up to 248.4 ~ 22,069.9 times that in the 0.5 m shoot. The expression of *PeGT47–07283* and *PeAT25494* showed relatively small changes (3.2 ~ 5.2-fold) (Fig. 9).

**Fig. 10:**
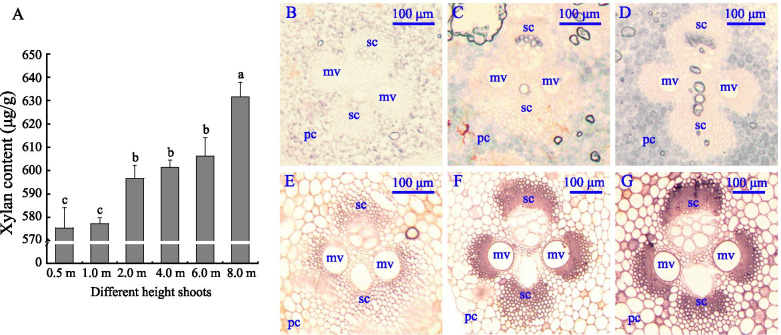
Xylan content and structural polysaccharides staining in different height shoots. **A**: Xylan content. Lowercase letters a, b, c, d and e indicate significant differences in expression level (*p* < 0.05). **B - G**: Structural polysaccharide staining intensity of vascular bundle transverse section in 0.5 m (**B**), 1.0 m (**C**), 2.0 m (**D**), 4.0 m (**E**), 6.0 m (**F**), 8.0 m (**G**) shoots. pc: parenchyma cells; sc: sclerenchymatous cell; mv: metaxylem vessel

To determine the connection between *PeGT43*s expression and the abundance of xylan, the xylan contents and structural polysaccharide staining intensity in the same tissues were measured. Xylan contents increased significantly during the growth of shoots, especially in 8.0 m shoots (Fig. 10), which was reasonable as *PeGT43*s were highly expressed in the older shoots. The increase in xylan contents in 2.0 m shoots may be due to the upregulated expression accumulation of some *PeGT43*s. The intensity of structural polysaccharide staining also increased with the growth of shoots (Fig. 10), which was consistent with the expression of *PeGT43*s.

### Subcellular localization analysis and validation of PeMYB35 binding 5′ UTR/promoter of *PeGT43–5* in yeast

**Fig. 11:**
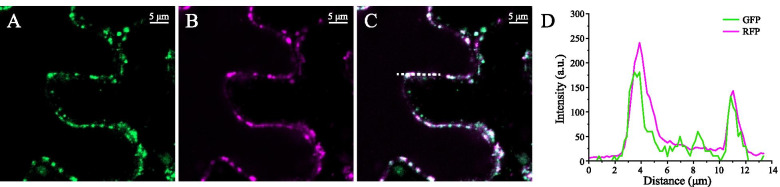
Subcellular localization of PeGT43–5 in *N. benthamiana* leaves expressed with *PeGT43–6*. **A - C**: Fluorescent localization of PeGT43–5 and PeGT43–6 fused in-frame to GFP (**A**), mRFP-tagged Golgi marker (**B**) and the merged images (**C**). **D**: Intensity plot of GFP and mRFP from the transfection shown in (**C**)

Correlation analysis based on the coexpression network and transcriptome data revealed that *PeGT43–5* and *PeGT43–6* had the strongest correlation with *PeMYB*s and *PeNAC*s. In addition, *PeGT43–5* was also highly positively correlated with *PeGT47–07283*, which may be relevant to the formation of glycosyltransferase polymers (Fig. 7 and Fig. S3). Biosynthesis of xylan occurs in the Golgi apparatus. Previous study shows that coexpression of *IRX9* and *IRX14* contributes to better Golgi localization results [15]. Therefore, the sequences encoding PeGT43–5 and PeGT43–6 (Table S9), which were orthologs of IRX9 and IRX14, were fused in-frame to the sequence encoding green fluorescent protein (GFP) at the 3′ end respectively and then cotransfected tobacco leaves with a mRFP-tagged Golgi marker together. The resulting overlaid signals suggested that they are Golgi-localized glycosyltransferases (Fig. 11).

**Fig. 12:**
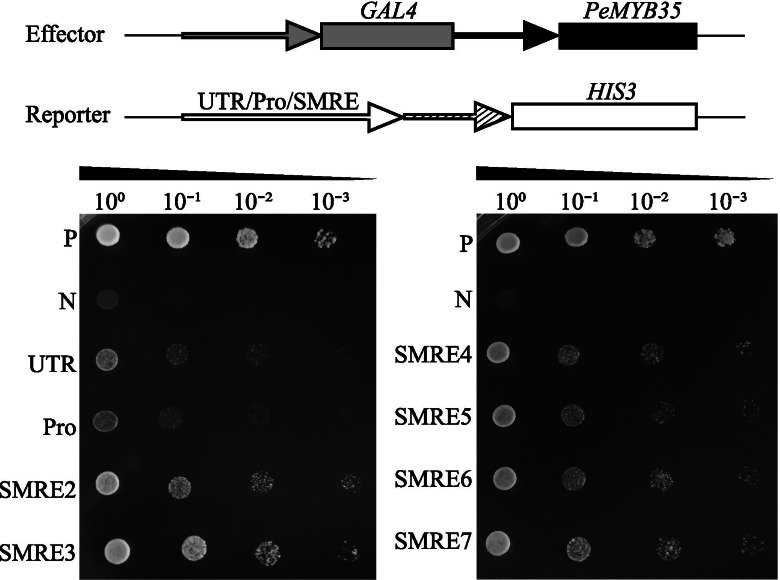
Y1H assay for the interaction between PeMYB35 and the 5′ UTR/promoter of *PeGT43–5*. The 5′ UTR/promoter of *PeGT43–5* as well as triple SMRE elements were inserted into pHIS2 vector. The reporter and effector constructs were co-transformed into yeast Y187 cells, and the transformed cells were identified by spotting serial dilutions of yeast onto selective medium SD/−Leu/−Trp (DDO) and SD/−Leu/−Trp/−His (TDO) containing 20 mmol·L^− 1^ 3-amino-1,2,4-triazole (3-AT). P: positive control (p53::HIS2 + pGADT7::53); N: negative control (p53::HIS2 + pGADT7::PeMYB35); Pro: promoter

AtMYB103, the ortholog of PeMYB35, has been shown to act as the regulator of cell wall biosynthesis [3]. To better understand the underlying regulatory mechanism of *PeGT43*s, we isolated the untranslated region (5′ UTR, 1119 bp) and promoter (2539 bp) of *PeGT43–5*, as well as *PeMYB35* (Table S9). Moreover, triple SMRE elements including SMRE2 (ACCAACT), SMRE3 (ACCAAAC), SMRE4 (ACCAACC), SMRE5 (ACCTAAT), SMRE6 (ACCAACC) and SMRE7 (ACCTAAC) were synthesized. All fragments were inserted into yeast expression vectors. Potential binding sites of *PeMYB35* in the 5′ UTR/promoter of *PeGT43–5* were analyzed using a yeast one-hybrid (Y1H) assay. The positive control and the experimental group grew well on TDO medium containing 3-amino-1,2,4-triazole (3-AT). The transformants harboring triple elements showed the best growth, followed by the transformants harboring the 5′ UTR and those harboring the promoter (Fig. 12). In contrast, the negative control could not grow on the nutritional screening medium. The reason that transformants harboring a 5′ UTR grew better than those harboring a promoter might be the double tandem repeat of SMRE4 in the 5′ UTR. These experimental results indicated that PeMYB35 could bind the 5′ UTR/promoter of *PeGT43–5*, which suggests that PeMYB35 could regulate the expression of *PeGT43–5*.

## Discussion

Xylan is the major hemicellulose in both primary and secondary cell walls. Since GT43 is one of the important glycosyltransferase families contributing to xylan biosynthesis, exploration of the GT43 genes in bamboo will potentially provide insight to modify the biomass composition of bamboo suitable for biofuel production. Genome-wide identification of GT43 genes from five bamboo species revealed that GT43s in moso bamboo were the most abundant, indicating that woody bamboo had more GT43s than herbaceous bamboo. Only 12 GT43 genes were identified in *B. amplexicaulis*. This lower-than-expected number is likely due to the lower quality assembly of the *B. amplexicaulis* genome (848 Mb of the scaffold with an N50 length of 1.76 Mb) compared to that of moso bamboo (1886 Mb of the scaffold with an N50 length of 79.90 Mb) [5, 31]. There was a correlation between biological characteristics and the number of related genes. Monocots have more GT43 genes and a higher percentage of xylan in hemicellulose than dicots [9, 10]. Therefore, GT43 may be one of the factors affecting xylan biosynthesis. Collinearity between moso bamboo and rice provided evidence for the gene duplication of *PeGT43*s. The divergence time of the four pairs of *PeGT43*s was approximately50 ~ 60 million years ago (Mya) and that of the two pairs of *PeGT43*s was approximately10 Mya, which is similar to the time of the WGD events that occurred in moso bamboo [33].

The type and distribution of conserved motifs in GT43 proved the reliability of subfamily classification. GT43s from the same subfamily shared the same conserved motifs and those from different subfamilies possessed unique motifs, such as Motif 12 for the GT43-A subfamily and Motif 10 and Motif 14 for the GT43-B subfamily. There were differences between conserved motifs of GT43s from monocots and dicots. For example, Motif 14 and Motif 13 were specific to GT43-B and GT43-C subfamily members from monocots, respectively, whereas there were no specific motifs in dicots. In addition, different conserved motifs possessed various sugar donor and sugar acceptor sites, which presumably represents the functional differentiation of GT43s caused by conserved motif differentiation. Differentiated motifs of GT43s may function to recognize different glycosyl and xylan backbones [34, 35].

The expression of GT43 showed significant spatial and temporal specificity. *PeGT43*s were highly expressed in shoots and showed an increasing expression trend with the growth of shoots, which was consistent with the expression trend of cell wall related genes [6]. The cellulose and lignin contents in the RE section of moso bamboo shoots were significantly higher than those in the SD and RD sections [36]. The variations in the expression of *PeGT43*s and the contents of these components suggested the changes related to cell wall biosynthesis during rapid cell elongation. Moso bamboo shoot internodes can elongate rapidly but not thicken in diameter during growth and development. Thus, the formation of nodes of moso bamboo shoots is of great biological importance in supporting rapid upright growth. Genes highly expressed in the MIN were mainly enriched in secondary cell wall biosynthesis pathways such as the phenylpropane biosynthesis pathway. Genes preferentially expressed in the MNO were enriched in cell wall organization and membrane metabolism pathways [37]. The expression of *PeGT43*s in the MIN and MNO was higher than that in the YIN and YNO. The preferential expression of *PeGT43*s in the RE sections and nodes reflected their role in xylan biosynthesis adapted to the rapid growth of moso bamboo.

There are a set of MeJA-responsive elements in the *PeGT43*s promoter. MeJA plays an important role in plant self-defense as a responsive signal. For example, the exogenous application of MeJA increased the total free phenolics, ferulic acid, caffeic acid, coumaric acid, and chlorogenic acid in the leaves of bread wheat. Upregulation of phenylpropanoid cascades in response to exogenous application of MeJA which reduces cell wall disruption and tissue disintegration and increases suberization and lignification of the plant cell may increase the resistance to spot blotch [38]. Genes for pectin methylesterase (PME), endo-polygalacturonase (PG), glycosyltransferase (GT) and cellulose synthase (CesA) are also significantly upregulated by MeJA, suggesting that MeJA treatment not only promotes the degradation of polysaccharides to destroy the cell wall but also promotes the biosynthesis of new polysaccharides [39]. In addition, cellulose, hemicellulose, and pectin polysaccharides induced by MeJA act as damage-associated molecular patterns (DAMPs) to activate plant innate immunity [40]. The MeJA-responsive and wound-responsive elements in the *PeGT43*s promoter suggest that *PeGT43*s may be involved in hormone signaling pathways of moso bamboo.

Xylan, as a linkage between cellulose and lignin, increases in content with the growth of bamboo shoots, which was similar to the content changes of other cell wall components [41]. This result implied that *PeGT43*s are inseparably related to other genes involved in cell wall biosynthesis [9]. In this study, the coexpression network of *PeGT43*s included genes encoding CESAs, GHs, COBLs and ATs. CESAs and COBLs are associated with cellulose accumulation, such as CESAs involved in the biosynthesis of cellulose and COBLs in the polar arrangement of cellulose to affect the toughness and stiffness [42, 43]. Protein–protein interactions have been shown to constitute an important organizing principle for xylan biosynthetic enzymes [44]. Genes from the GT43 and GT47 families are assumed to form complexes in the Golgi apparatus involved in xylan backbone biosynthesis [45]. Wheat (*Triticum aestivum*) GTs were proposed to synthesize arabinoxylan via a glucurono(arabino)xylan synthase complex form. The complexes of TaGT43–4, TaGT47–13 and TaGT75–4 obtained by immunoprecipitation were characterized by xylosyltransferase, glucuronosyltransferase and arabinosyltransferase activities, respectively [46, 47]. In this study, the significant correlation between *PeGT43*s and genes from the GT47 family may be related to complex formation in moso bamboo, which needs to be further validated.

GHs are involved in the degradation of cell wall polysaccharides [48] and induce lignin content changes and biosynthesis of cellulose during seed maturation [49], considering their coexpression with *GT*s and *CESA*s. *OsGH9A3* and *OsGH9B5* are coexpressed with *OsCESA1*, *OsCESA3* and *OsCESA8* and involved in primary cell wall cellulose synthesis [50]. ATs are involved in xylan biosynthesis by adding acetyl to xylan chains [11]. OsTBL1 and OsTBL2 are ATs that acetylate xylan in rice. They are responsible for acetylation of both xylan main and side chains and their mutants display a stunted growth phenotype with varying degrees of dwarfism [51]. Furthermore, the expression patterns of *PeGH*s and *PeAT*s are similar to those of *PeGT43*s, suggesting their synergistic role in cell wall remodeling.

*PeMYB35* and *PeMYB37*, coexpressed with *PeGT43*s, are homologs of *AtMYB103*, which play a critical role in secondary wall biosynthesis in *A. thaliana* [52]. *PeMYB35* and *PeMYB37* were expressed at low levels in 0.2 m shoots and increased substantially in 1.5 m and 3.0 m shoots, which was consistent with the expression trend of *PeGT43*s. Additionally, qRT–PCR showed that the relative transcript abundance of most *PeGT43*s showed a significant increase in 4.0 m shoots, along with high expression of *PeMYB35*. Based on these results, it is speculated that the expression level changes of *PeMYB*s and *PeNAC*s, especially *PeMYB35*, may contribute to the regulation of *PeGT43*s.

Furthermore, there are abundant MYB-binding elements on the promoter of *PeGT43*s, which belonged to the SMRE motif. SMRE elements are involved in the lignin, cellulose and hemicellulose biosynthetic pathways by binding to MYB, NAC and KNAT transcription factors [28, 53]. In Arabidopsis, AtMYB83 increas the expression level of *AtIRX14L* significantly [28]. NAC and MYB in *Mangifera* have also been found to bind to the promoters of *MlGT43A* and *MlGT43B* [29]. In this study, the binding of PeMYB35 to the 5′ UTR/promoter of *PeGT43–5* with different SMRE elements was verified in yeast, suggesting that PeMYB35 might play a regulatory role in the expression of *PeGT43*s for xylan biosynthesis. In addition, GT43 genes in other species like IRX9 and IRX14, as well as transcription factors that bind to the GT43 gene promoters, are related to xylan biosynthesis. Similarly involved in xylan biosynthesis, *PeIRX10* has been identified in moso bamboo to have the ability to complement the deficiency of *irx10* in Arabidopsis, which suggests a functional similarity between *PeIRX10* and *irx10* [54]. Therefore, it is assumed that *PeGT43*s are related to xylan biosynthesis as well. However, the content and structure of xylan differs in monocots and dicots. Considering the rapid growth characteristics of moso bamboo and gene duplication of *PeGT43*s, the functions of *PeGT43*s in moso bamboo require additional experimental evidence.

## Conclusions

GT43s are essential for xylan biosynthesis and function in cell wall modification. A total of 17 *PeGT43*s belonging to three subfamilies were identified in the moso bamboo genome. The *cis*-acting elements involved in MeJA, ABA and wound response, as well as MYB-binding sites were present in the promoters of *PeGT43*s. Transcriptome data revealed that *PeGT43*s were highly expressed in shoots, especially in rapid elongation potions and nodes. The expression of *PeGT43*s and their coexpressed genes associated with cell wall biosynthesis was consistent with the xylan content and structural polysaccharide staining intensity in bamboo shoots. In addition, Y1H assay confirmed that PeMYB35 could bind the 5′ UTR/promoter of *PeGT43–5*, indicating the regulatory relationship between TFs and *PeGT43*s in moso bamboo. These results lay a foundation for further investigation of *PeGT43*s function in the biosynthesis of xylan during rapid growth of moso bamboo.

## Methods

### Plant materials

Representative sections (the fifteenth section from the base upwards) of moso bamboo shoots of different heights (0.5 m, 1.0 m, 2.0 m, 4.0 m, 6.0 m and 8.0 m) were collected with permission from the bamboo forest experimental site of Jiangxi Academy of Forestry located in Nanchang, Jiangxi Province, China. The bamboo samples were frozen in liquid nitrogen and stored at − 80 °C before RNA extraction and determination of xylan contents. Three biological replicates were used for each experiment.

### Identification of GT43 genes in bamboo

The GT43 proteins of *Brachypodium distachyon* (Brachypodium), *Oryza sativa* (rice), *Zea mays* (maize), *Arabidopsis thaliana* (Arabidopsis), *Gosspium hirsutum* (cotton) and *Populus trichocarpa* (poplar) from the Carbohydrate-Active Enzyme (CAZY) database (http://www.cazy.org/) were selected as the query to search the protein sequences of GT43 family members from the bamboo genome database BambooGDB (http://bamboo.bamboogdb.org/) and other Bambusoideae (*Olyra latifolia*, *Raddia guianensis*, *Raddia guianensis*, *Bonia amplexicaulis*) databases (http://www.genobank.org/bamboo) [30]. The sequences were considered as predicted GT43s if the sequences had E-values of ≤ − 10 and intact glycosyltransferase 43 domains. Molecular characteristics were calculated by the online program ExPasy (http://www.expasy.org/tools/) [55].

### Phylogenetic and structure analysis

Phylogenetic analysis was conducted according to the maximum likelihood method (1000 bootstrap replicates) of the MEGA X software. The conserved motifs were identified using the Multiple EM for Motif Elicitation program v5.4.1 (https://meme-suite.org/meme/tools/meme) [56] and visualized with TBtools [57]. Collinear regions between *PeGT43*s and the *Ka* (nonsynonymous substitution) / *Ks* (synonymous substitution) ratio between the paralogous pairs were analyzed by MCScanX [57]. The divergence time of the duplication event was estimated with the rate λ = 6.5 × 10^− 9^ in monocots [58].

### Expression pattern and co-expression network

Upstream sequences (2000 bp) of the *PeGT43*s start codon were selected as a promoter to predict hormone, abiotic stress response elements and transcription factor binding elements using PlantCARE online program (http://bioinformatics.psb.ugent.be/webtools/plantcare/html/) [59]. Potential regulation was predicted using PlantRegMap/PlantTFDB v5.0 (http://plantregmap.gao-lab.org/) [60]. Tissue expression analysis of *PeGT43*s was measured as fragments per kilobase of exon model per million mapped reads (FPKM) and transcripts per million (TPM) based on transcriptomic data of 26 different tissues, shoots of different heights (0.2 m, 0.5 m, 1.0 m, 2.0 m, 3.0 m, 5.0 m, 6.0 m, 7.0 m), the sections of start of division (SD), rapid division (RD) and rapid elongation (RE) of shoots, and the shoot apical meristem (SAM), young internode (YIN), young node (YNO), mature internode (MIN) and mature node (MNO) of shoots [31, 36, 37, 61]. Genes coexpressed with *PeGT43*s were analyzed by bamboonet (http://bioinformatics.cau.edu.cn/bamboo/index.html) [62] with a single direction rank of Pearson correlation coefficient (PCC) and mutual rank (MR) score. Correlation analysis of expression levels between *PeGT43*s and transcription factors was performed by OmicShare online tools (https://www.omicshare.com/tools/) based on PCC.

### qRT-PCR, xylan contents and structural polysaccharides staining analysis

Total RNA was isolated using TRIzol reagent (Invitrogen, USA). First-strand cDNA was synthesized with 1 μg RNA using a PrimeScript™ RT Reagent Kit (TaKaRa, Japan) according to the manufacturer’s instructions. qRT–PCR experiments were performed using SYBR Green chemistry (Roche, Mannheim, Germany) on a qTOWER 2.2 system (Analytik, Jena, Germany) according to the manufacturer’s directions. Gene-specific primers were designed by Primer Premier 5.0 software (Table S7), and *PeTIP41* was selected as an internal control [63]. Three biological replicates and three technical replicates were employed in each experiment. The final relative expression levels were calculated using the 2^-△△Ct^ method [64]. The xylan contents were measured based on the orcinol-HCl method [65] with a commercial kit (item number S0169O-1) from the Kete Biotechnology Company of Jiangsu, China. Structural polysaccharide staining was performed by the zinc iodide chloride method.

### Subcellular localization and yeast one-hybrid assay

The coding sequences of *PeGT43–5* and *PeGT43–6* were cloned and fused in frame with that of GFP in pCAMBIA1300 vector, respectively. The resulting constructs were cotransformed together into tobacco leaves with a construct for expression of the Golgi marker mRFP. The fluorescence signals were recorded with a confocal laser scanning microscope (LSM 980; Zeiss) after 3 days. Each inoculation was performed on at least triplicate leaves.

Yeast one-hybrid (Y1H) assays were performed to detect whether PeMYB35 (PH02Gene26889) is bound to the untranslated region (5′ UTR) and promoter of *PeGT43–5*. The 5′ UTR/promoter sequence as well as triple repeated SMREs were cloned into pHis2. In addition, the coding sequence of *PeMYB53* was cloned into pGADT7-Rec2. The combinations of pPeGT43–5′ UTR/promoter::His2 with pGADT7::PeMYB53, pSMRE4::His2 with pGADT7::PeMYB53, and pSMRE6::His2 with pGADT7::PeMYB53 were cotransformed into the Y187 yeast strain. The transformed yeasts were cultured on DDO medium (−Trp/−Leu) for 3 days and then transferred to DDO medium (−Trp/−Leu/−His) plates containing20 mmol·L^− 1^ 3-amino-1,2,4-triazole (3-AT) for 4 days. The cotransformants containing p53::His2 with pGADT7::53 and p53::His2 with pGADT7::PeMYB35 were selected as the positive control and negative control, respectively. The primers required for Y1H and subcellular localization analysis are shown in Table S8.

## Supplementary Information

**Additional file 1: Table S1.** Basic characteristics of proteins encoded by GT43 genes in five bamboo species.

**Additional file 2: Table S2.** *Ka / Ks* and divergence time analysis of PeGT43s pairs.

**Additional file 3: Table S3.** The *cis*-acting elenment in the promoter of *PeGT43s*.

**Additional file 4: Table S4.** The potential transcription factors binding to the promoter of *PeGT43s*.

**Additional file 5: Table S5.** The expression of *PeGT43s* in different tissues based on transcriptome data.

**Additional file 6: Figure S1.** Expression profiles of *PeGT43s* in different height shoots of moso bamboo.

**Additional file 7: Figure S2.** Gene Ontology classification of genes coexpressed with PeGT43s.

**Additional file 8: Table S6.** Cell wall-associated genes (PCC > 0.8) used for co-expression network construction.

**Additional file 9: Figure S3.** Correlation analysis of *PeGT43s* and co-expressed genes.

**Additional file 10: Table S7.** Primers used for RT-qPCR.

**Additional file 11: Table S8.** Primers used for Y1H expression vector construction.

**Additional file 12: Table S9.** The coding sequences and upstream sequences isolated in this study.

## Data Availability

The datasets supporting the conclusions of this article are included within the article and its additional files. The genomics data of moso bamboo and other Bambusoideae are available in BambooGDB database (http://bamboo.bamboogdb.org) and The Germplasm Bank of Wild Species database (http://www.genobank.org/bamboo), respectively. The genomics data of *Brachypodium distachyon* (Brachypodium), *Oryza sativa* (rice), *Zea mays* (maize), *Arabidopsis thaliana* (Arabidopsis), *Gosspium hirsutum* (cotton) and *Populus trichocarpa* (poplar) are available in the Phytozome database (https://phytozome-next.jgi.doe.gov). The public RNA-seq data are available at NCBI Sequence Read Archive (SRA) database (https://www.ncbi.nlm.nih.gov/sra) under the accession number SRR5091764 ~ SRR5091815, SRR6171235 ~ SRR6171258, SRR9218100 ~ SRR9218108, SRR4209871 ~ SRR4209890.

## Abbreviations

3-AT: 3-amino-1,2,4-triazole
ABA: Abscisic acid
AT: Acetyltransferase
CesA: Cellulose synthase
COBL: Glycosylphosphatidylinositol-anchored protein-like
DAMP: Damage-associated molecular pattern
DDO: Double Dropout Supplements
FPKM: Fragments per kilobase per million
GA: Gibberellins
GH: Glycosyl hydrolase
GlcUA: α-D-glucuronic acid
GT: Glycosyltransferase
GX: Glucuronoxylan
IRX: IRREGULAR XYLEM
MeJA: Methyl jasmonate
Mya: Million years ago
PCC: Pearson correlation coefficient
PG: Endo-polygalacturonase
PME: Pectin methylesterase
qRT–PCR: Quantitative Real-time PCR
TDO: Triple Dropout Supplements
TFs: Transcription factors
TPM: Transcripts per million
Y1H: Yeast one-hybrid
