# Supplementary material for: Identification and expression analysis of the glycosyltransferase GT43 family members in bamboo reveal their potential function in xylan biosynthesis during rapid growth
Source: BMC Genomics. 2021 Dec 2;22:867. doi: 10.1186/s12864-021-08192-y (PMC8638195; doi:10.1186/s12864-021-08192-y)
Supplement: Supplementary file 6 — Additional file 6: Figure S1. Expression profiles of PeGT43s in different height shoots of moso bamboo. [file 12864_2021_8192_MOESM6_ESM.docx]

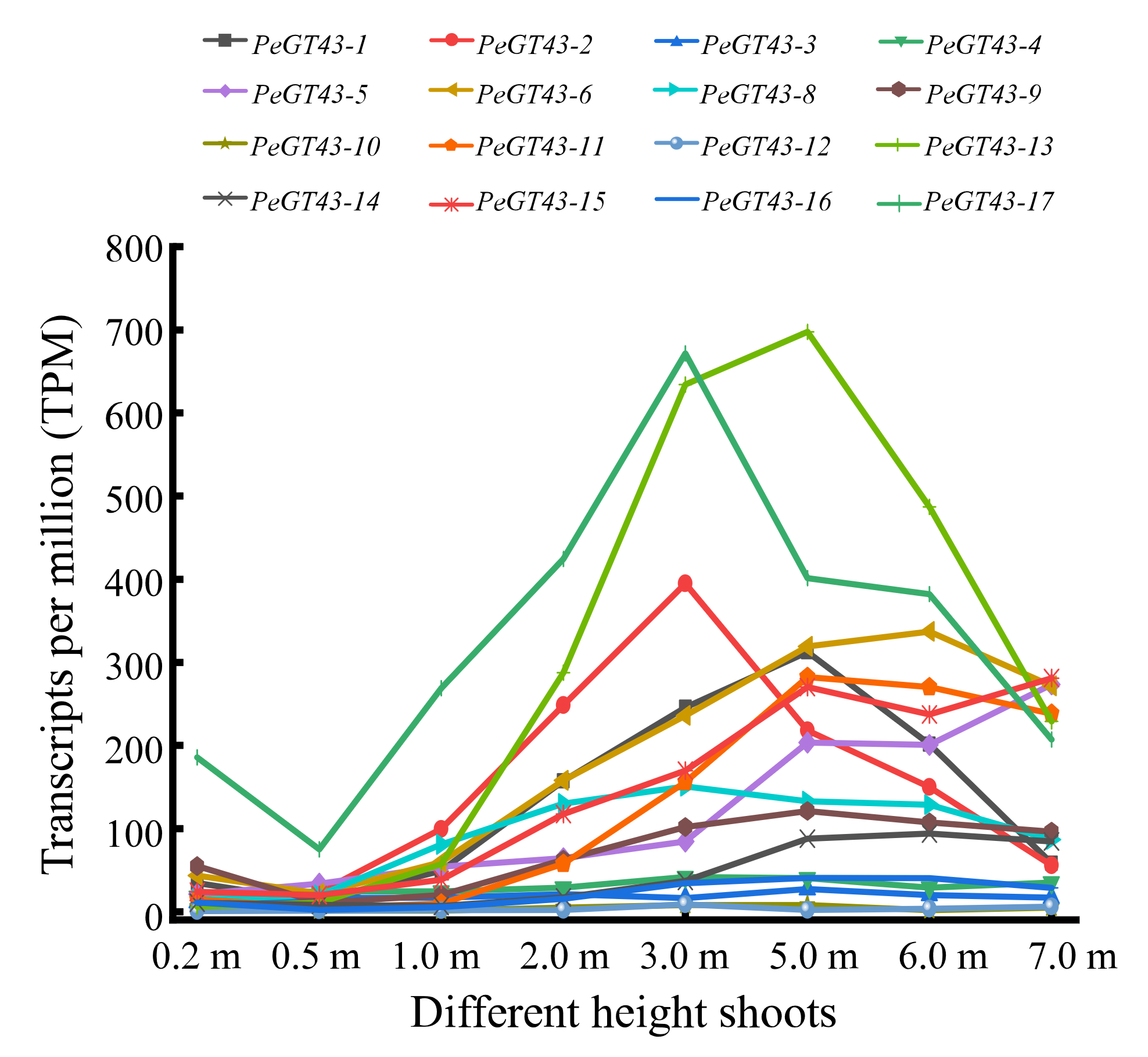


**Figure S1.** Expression profiles of *PeGT43*s in different height shoots of moso bamboo. Expression patterns of *PeGT43*s with significant transcriptional changes were showed in folding line chart, using the transcripts per million (TPM) as the value of the vertical coordinate. The TPM value was listed in Table S5.
