# Supplementary material for: Identification and expression analysis of the glycosyltransferase GT43 family members in bamboo reveal their potential function in xylan biosynthesis during rapid growth
Source: BMC Genomics. 2021 Dec 2;22:867. doi: 10.1186/s12864-021-08192-y (PMC8638195; doi:10.1186/s12864-021-08192-y)
Supplement: Supplementary file 7 — Additional file 7: Figure S2. Gene Ontology classification of genes coexpressed with PeGT43s. [file 12864_2021_8192_MOESM7_ESM.docx]

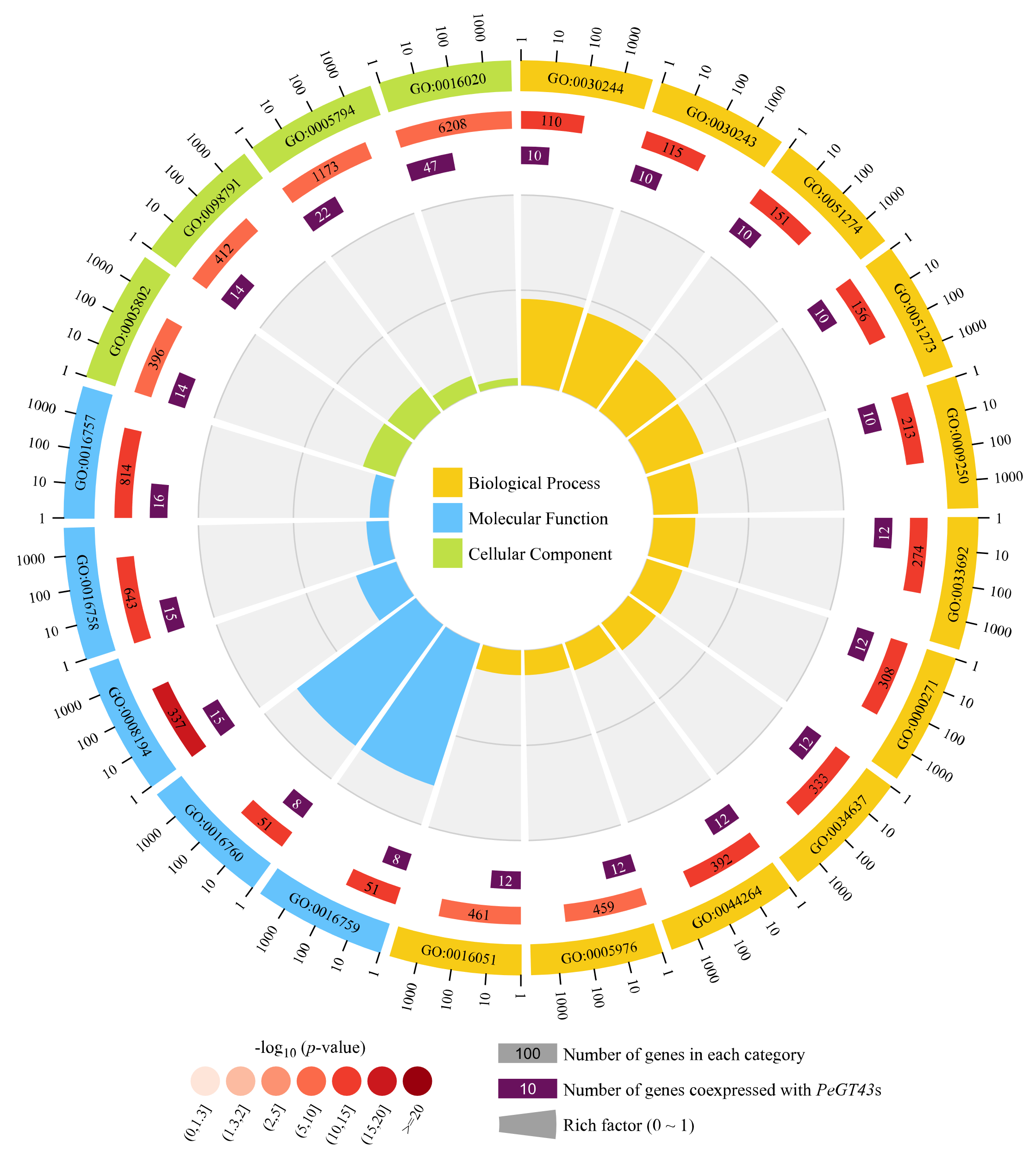


**Figure S2.** Gene Ontology classification of genes co-expressed with *PeGT43*s. Outside the circle indicates the scale of the gene numbers.
