# Supplementary material for: Identification and expression analysis of the glycosyltransferase GT43 family members in bamboo reveal their potential function in xylan biosynthesis during rapid growth
Source: BMC Genomics. 2021 Dec 2;22:867. doi: 10.1186/s12864-021-08192-y (PMC8638195; doi:10.1186/s12864-021-08192-y)
Supplement: Supplementary file 9 — Additional file 9: Figure S3. Correlation analysis of PeGT43s and co-expressed genes. [file 12864_2021_8192_MOESM9_ESM.docx]

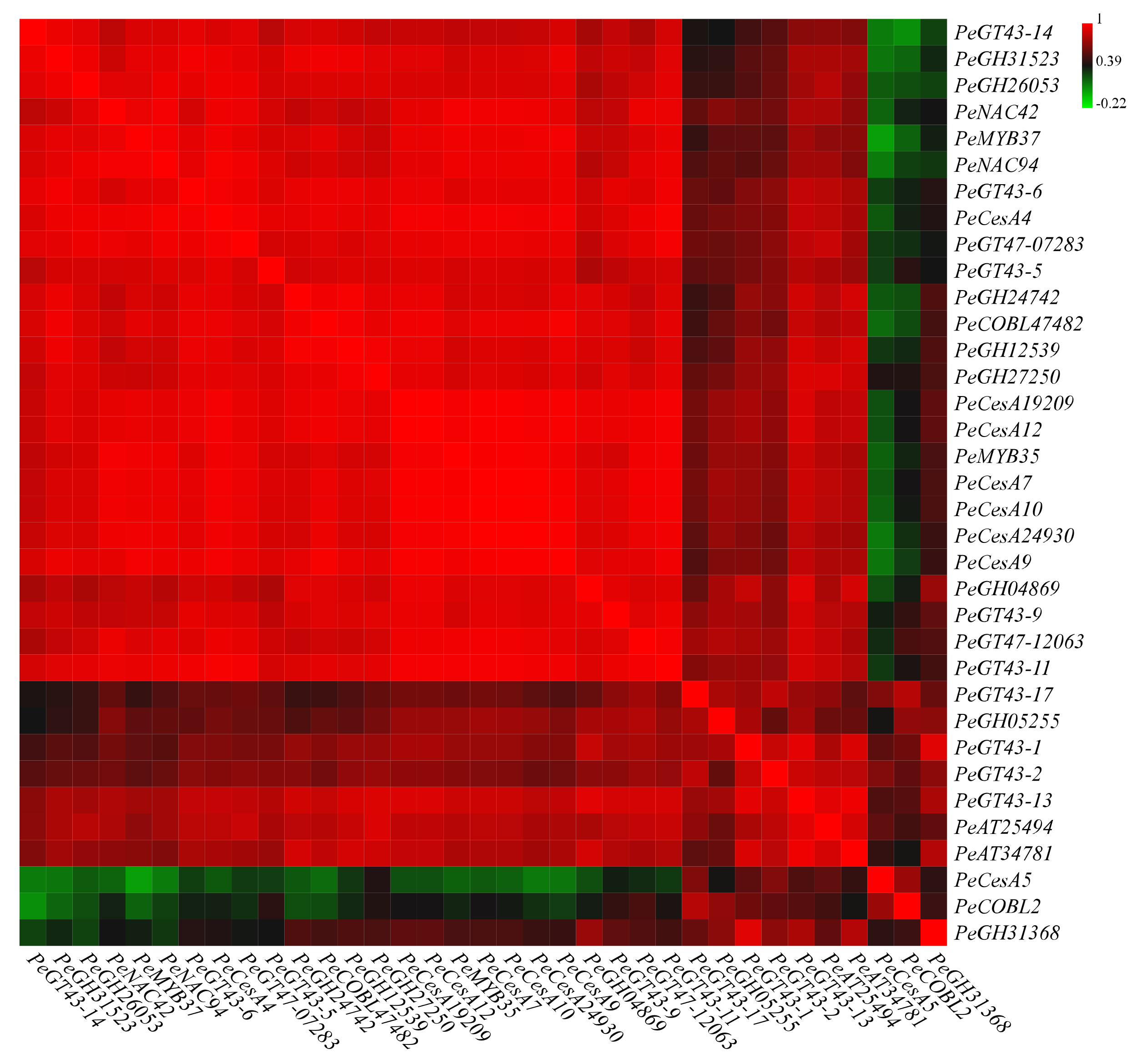


**Figure S3.** Correlation analysis of *PeGT43*s and co-expressed genes. The color bar indicates Pearson correlation coefficient.
